# Doctor’s burnout and interventions

**DOI:** 10.1017/ipm.2020.32

**Published:** 2020-05-13

**Authors:** Claire Kehoe, Elizabeth Barrett

**Affiliations:** The Lucena Clinic Rathgar, Dublin, Republic of Ireland, (Email: claire.kehoe@UCD.ie); University College Dublin, Children Health Ireland, Dublin, Republic of Ireland

Dear Editor,

Recent work by Niamh Humphries *et al*. has shown ongoing trends for emigration and implicated several factors. While migration has always been a part of medical training, there is increasing concern about professionals leaving Ireland without intending to return (December 2019 by Niamh Humphries *et al*.). This worrying phenomenon has previously been attributed to budget cuts and deteriorating working conditions in the Irish Health System (Humphries *et al*. 2015, 2019), ‘in a system that is overstressed, understaffed and has ever worsening morale’. In Ireland, the ‘National study of wellbeing of hospital doctors in Ireland’ by Hayes in 2017 reported that one-third of respondents reported burnout; four-fifths, significant work stress; and one-tenth, severe or extremely severe stress levels (Hayes *et al.* 2017). Given recent struggles in the Irish health services and particularly high rates of vacant posts in psychiatry, this area merits close consideration. A more recent publication in August 2018 by Margiota exploring burnout among consultant-level doctors in Irish context showed that 42% endorsed a high level of burnout (Margiota *et al.* 2018). Burnout was reported by staggering three-quarters of all Emergency staff in the Cork University Hospital in 2017 (Chernoff *et al.* 2018; Kelleher 2018), meaning there is an urgency to drive a country-wide approach towards reducing and preventing burnout.

‘Burnout’ has been defined in the updated WHO International Classification of Disease 11th Edition (ICD-11, May 2019), as an ‘occupational phenomenon… a syndrome resulting from chronic workplace stress that has not been successfully managed’. It is characterised by three components: exhaustion; cynicism, increased mental distance from one’s job; and reduced professional efficacy (WHO 2019). Burnout is most commonly understood to occur in high-demand and low resource-environments, with disconnection between the workers’ expectations and experiences, and neglect of the individual’s needs (Lemaire et al. 2017; Ruzycki *et al.* 2018).

Burnout is an international concern. The Medical Protection Society issued a recent guide on ‘Breaking the burnout cycle. Keeping Doctors and Patients Safe’ following an online survey of international doctors. Forty-seven per cent of doctors start their working day feeling tired and 30% are unable to take a break during their working day (Medical Protection 2019). West wrote in the Lancet in 2016 that American physician burnout has reached ‘epidemic’ proportions affecting patients’ care, physicians’ own care and safety, professionalism, and healthcare systems’ viability (West *et al.* 2016). Burnout can present across professional life, and indeed in medical students. In a large collaborative study of psychiatric trainees in 22 countries in 2010, on average 37% of trainees experienced burnout, with Ireland scoring higher at 39% (Jovanovic *et al.* 2016). Burnout seemed to impact an astounding 50% of American doctors, more severely in the ‘front-line specialties’ – general internal medicine, family medicine, emergency medicine and neurology (Shanafelt *et al.* 2015). Risks factors for burnout vary – ‘younger age’, ‘female gender’, ‘not having children’, ‘long working hours’, ‘not having regular time to rest’, ‘lack of clinical supervision’, and in the trainee study, career choice seemed to impact – ‘not opting for psychiatry as a first career choice’ (Amoafo *et al.* 2015; Bianchi *et al.* 2018).

Why does it matter to Irish Psychiatry? Garman *et al*. surveyed clinicians and patients’ perception of care, in community mental health services, and higher burnout was associated with reduced patients’ satisfaction with autonomy, treatments and therapists (Garman *et al.* 2002). Moreover, De Jong *et al*. in 2016 revealed that doctors’ burnout, depression and fatigue were associated with a significant increase in medical incidents (De Jong *et al.* 2016).

From a mental health perspective, physicians generally have been shown to be slow to seek supports. Hayes *et al*. found that two-thirds of hospital doctors would not want others to know they had mental health issues (Hayes *et al.* 2017). Most had never sought help for burnout or depression (64%), and 10% even considered leaving medicine due to extreme burnout (Hayes *et al.* 2017). Burnout has been found to be strongly associated with depression, suicidal ideation and suicide (Hayes 2018; Jovanovic *et al.* 2019; Kane 2019). In the 2019 follow-up of the International Trainee Burnout Study in 22 countries, 15% of trainees were depressed, 12% presented with suicidal ideation and 0.7% had attempted suicide (Jovanovic *et al.* 2019). Similarly, in ‘The National Physician Burnout, Depression, and Suicide Report’, 11% of burnout physicians had at least one symptom of depression, 4% full clinical depression, 14% suicidal ideation and 1% had attempted suicide (Kane 2019).

What can we do in Irish Psychiatry? In view of the above, we can argue that interventions are urgently required at systemic levels.

Firstly, at an individual level, there is evidence that doctors are OFTEN/USUALLY responsible and aware of these issues – they are shown to manage, or attempt to manage, with positive coping mechanisms – exercise (48%), family/friends time (43%), sleep (39%) and listening to music (33%). Less helpful mechanisms are also evident – isolation (41%), junk food (32%), alcohol (23%) or cannabis (1%) (West *et al.* 2016).

Secondly, supportive organisational actions should be included in any hospital culture (Shanfield *et al.* 1985; West *et al.* 2016). Institutional approaches need to move away from merely focusing on individual physicians towards fostering systems that support clinicians in the face of work stressors. For instance, burnout levels were significantly reduced from 54% to 44%, when mindfulness, stress management, reduction of working hours, mentoring schemes, recognition of extra-clinical work and small group discussions were available (West *et al.* 2016).

Specific, evidenced interventions have utility on the ground and improve understanding and communication. Groups like ‘Schwartz rounds’ support hospital-wide communication sessions, foster positive interdisciplinary work and impact positively on staff satisfaction (Mayben 2018; Silke *et al.* 2019); or ‘Balint groups’: small professional reflective practice groups aiming to validate emotional experiences, also positively received (Balint 1957; Douglas *et al.* 2016; Hind 2018). These groups are now being evidenced in Ireland and Schwartz Rounds have piloted (Silke *et al.* 2019). The College of Psychiatrists of Ireland and the Irish College of General Practitioners have both already included reflective practice groups in their curriculum for trainees (Irish College of General Practitioners 2016; College of Psychiatrists of Ireland 2018). Finally, the ‘National Study of Wellbeing of Hospital Doctors in Ireland’ report (Hayes *et al.* 2017) recommended some systemic interventions: (i) priority to be given to staff welfare. Given our clinical roles, it is perhaps surprising that we do not support ourselves or our colleagues in this regard and then, how effectively can we support these conversations with our patients? (ii) recognition of occupational stress, (iii) robust support of appropriate educational and training needs and (iv) integration of doctors in management structures – widely absent in some spheres in the health services. In contrast to some other countries, it is striking that in Ireland, the need for protected time for clinicians’ self-care activities is not recognised contractually (Viney *et al.* 2015).

Although it is well and good to devise specific interventions, it should be stressed that under-resourcing remains a major factor for burnout (West *et al.* 2016). No clinician can use individual or systemic approaches, from mindfulness to Balint groups, to tackle distress caused by understaffing. Should training bodies and organisations such as the Irish Medical Council take a leading role in supporting robust staffing levels and resourcing, in the interests of both doctors and patients given the impact of burnout?

To address health staff’s issues, the Irish Health Service Executive created in April 2018 a new unit dedicated to staff welfare ‘Workplace Health and Wellbeing Unit’ (HSE Work Well Human Resource Services 2017). We are keen to see outputs from this service. We suggest that systemic approaches with greater evidence be supported in the first instance for both trainees and consultants – and indeed wider multidisciplinary teams across healthcare.

## References

[ref1] Amoafo E , Hanbali N , Patel A , Singh P (2015). What are the significant factors associated with burnout in doctors? Occupational Medicine (London) 65, 117–121. 10.1093/occmed/kqu144 25324485

[ref2] Balint M (1957). The Doctor, His Patient and the Illness. Churchill Livingstone: London.

[ref3] Bianchi R , Schonfeld I , Laurent E (2018). Burnout syndrome and depression: understanding depression In Clinical Manifestations, Diagnosis and Treatment (ed. Yong-Ku Kim ), Vol. 2, pp. 187–202. Springer Nature: Singapore.

[ref4] Chernoff P , Adedokun C , O’Sullivan I , McManus J , Payne A (2018). Burnout in the Emergency Department hospital staff at Cork University Hospital. Irish Journal of Medical Science 46, 667–674. 10.1007/s11845-018-1871-5 30051165

[ref5] College of Psychiatrists of Ireland (2018). Regulations for basic and higher specialist training in psychiatry. July 2012 Revision 6 July 2018.

[ref6] De Jong MA , Nieuwenhuijsen K , Sluiter JK (2016). Common mental disorders related to incidents and behaviour in physicians, Occupational Medicine 66, 506–513. 27605575 10.1093/occmed/kqw030

[ref7] Douglas L , Feeney L (2016) An established practice in new surroundings: concepts, challenges, pitfalls and guidelines for NCHD Balint Groups. Irish Journal of Psychological Medicine 34, 1–5. 10.1017/ipm.2015.63 30115164

[ref8] Garman AN , Corrigan PW , Morris S (2002). Staff burnout and patient satisfaction: evidence of relationships at the care unit level. Journal of Occupational Health Psychology 7, 235–241. 12148955

[ref9] Hayes B (2018). Who’s looking after the doctor’s health? Stress and burnout in doctors. AGM 2018. The Irish Medical Organisation.

[ref10] Hayes B , Walsh G , Prihodova L (2017). National study of wellbeing of hospital doctors in Ireland. Royal College of the Physicians of Ireland. April 2017.

[ref11] Hind D (2017). Balint groups in undergraduate medical education: a systematic review (2017). Psychoanalytic Psychotherapy. December 2017.

[ref12] HSE Work Well Human Resource Services (2017). Strategy for doctors’ health and wellbeing 2018–2021. ISBN NUMBER: 978-1-78602-072-7.

[ref13] Humphries N , Connell J , Negin J , Buchan J (2019). Tracking the Leavers: towards a better understanding of doctor migration from Ireland to Australia 2008–2018. Human Resources for Health 17, 36. 31138211 10.1186/s12960-019-0365-5 PMC6540407

[ref14] Humphries N , McAleese S , Matthews A , Brugha R (2015). ‘Emigration is a matter of self-preservation. The working conditions … are killing us slowly’: qualitative insights into health professional emigration from Ireland. Human Resources for Health 13, 35. 25981629 10.1186/s12960-015-0022-6 PMC4437248

[ref15] Irish College of General Practitioners (2016). ICGP curriculum for gp training in Ireland.

[ref16] Jovanovic N , Beezhold J , Tateno M , Barrett E , Vlachos I , Fiorillo A , et al. (2019). Depression and suicidality among psychiatric residents – results from a multi-country study. Journal of Affective Disorders 249, 192–198. 30772747 10.1016/j.jad.2019.02.023

[ref17] Jovanovic N , Podlesek A , Volpe U , Barrett E , Ferrari S , Rojnic Kuzman M , et al. (2016). Burnout syndrome among psychiatric trainees in 22 countries: risk increased by long working hours, lack of supervision, and psychiatry not being first career choice. European Psychiatry 32, 34–41. 26802982 10.1016/j.eurpsy.2015.10.007

[ref18] Kane L (2019). The national physician burnout, depression and suicide report. Medscape. January 16, 2019.

[ref19] Kelleher L (2018). Three quarters of CUH Emergency Staff suffer burnout. The Irish Examiner. August 7, 2018.

[ref20] Lemaire JB , Wallace JE (2017). Burnout among doctors. *British Medical Journal* 358, j3360. 10.1136/bmj.j3360 28710272

[ref21] Margiota F , Crudden G , Byrne D , Doherty AM (2018). Prevalence and co-variates of burnout in consultant hospital doctors: burnout in consultants in Ireland Study (BICDIS). Irish Journal of Medical Science 188, 355–364. 30128933 10.1007/s11845-018-1886-y

[ref22] Mayben J (2018). Enhancing compassion in relationships between staff and patients: a national evaluation of Schwartz Rounds in the UK 2014–2017. The King’s College London, February 20, 2018.

[ref23] Medical Protection (2019). Breaking the burnout Cycle. Keeping Doctors and Patients Safe. https://www.medicalprotection.org/ireland/breaking-the-burnout-cycle.

[ref24] Ruzycki SM , Lemaire JB (2018). Physician burnout. The Canadian Medical Association Journal 190, ES3. 10.1503/cmaj.170827 PMC5770253 29335264

[ref25] Shanafelt TD , Hasan O , Dyrbye LN , Sinsky C , Satele D , Sloan J , West CP (2015). Changes in burnout and satisfaction with work-life balance in physicians and the general US working population between 2011 and 2014. Mayo Clinic Proceedings 90:1600–1613. 26653297 10.1016/j.mayocp.2015.08.023

[ref26] Shanfield SB , Benjamin GAH (1985). Psychiatric distress in law students. *Journal of Legal Education* 35, 65–75.

[ref27] Silke A , Rushe H , Keating K , Thurstan R , Barrett E (2019). Caring for caregivers: an evaluation of Schwartz rounds in a paediatric setting. Irish Medical Journal 112, 951 31537055

[ref28] Viney R , Pace E (2015). The first 500 report. London Deanery Coaching and Mentoring Services *2008–*2010. https://www.yumpu.com/en/document/view/25225603/the-first-500-a-report-on-london-deanerys-coaching-and

[ref29] West CP , Dyrbye LN , Erwin PE , Tait D , Shanafelt TD (2016). Interventions to prevent and reduce physician burnout: a systematic review and meta-analysis. Lancet 388, 2272–2281. 27692469 10.1016/S0140-6736(16)31279-X

[ref30] World Health Organisation (2019). ICD-11th Edition, Revision of the International Classification of Diseases. https://www.who.int/mental_health/evidence/burn-out/en/.

